# Two-Component Signal Transduction Systems: A Major Strategy for Connecting Input Stimuli to Biofilm Formation

**DOI:** 10.3389/fmicb.2018.03279

**Published:** 2019-01-10

**Authors:** Cong Liu, Di Sun, Jingrong Zhu, Weijie Liu

**Affiliations:** School of Life Science, Jiangsu Normal University, Xuzhou, China

**Keywords:** biofilm, two-/three-/multi-component signal transduction systems, cross-regulation, input signals, c-di-GMP

## Abstract

Biofilms are multicellular communities of microbes that are encased within an extracellular matrix. Environmental factors induce bacteria to form biofilm. Bacteria have several regulatory mechanisms in response to environmental changes, and the two-component signal transduction system (TCS) is a major strategy in connecting changes in input signals to changes in cellular physiological output. The TCS employs multiple mechanisms such as cross-regulation, to integrate and coordinate various input stimuli to control biofilm formation. In this mini-review, we demonstrate the roles of TCS on biofilm formation, illustrating these input signals and modulation modes, which may be utilized by future investigations in elucidating the regulatory signals and underlying the mechanisms of biofilm formation.

## Introduction

Biofilms are common lifestyle, wherein bacteria grow as surface-associated multicellular communities (reviewed by Flemming and Wingender, 2010; Flemming et al., 2016). Biofilms are generally formed in response to stimuli that may be detrimental to bacterial growth, thus protecting themselves in adverse environments (reviewed by Hall-Stoodley et al., 2004). However, the natural environment is highly complex, and thus, it is difficult to identify the specific environmental factors that induce or inhibit biofilm formation. The two-component signal transduction system (TCS) is a major strategy of microbes in controlling their expression profiles in response to changes in the environment (Teschler et al., 2017; Xu et al., 2017). Conducting investigations on the influence of TCSs on biofilm formation have two advantages. First, the environmental factors that regulate biofilm formation can be identified by determining the input signals of these TCSs that are involved in the biofilm formation pathway (Stubbendieck and Straight, 2017; Camargo et al., 2018). Second, the regulatory mode of TCS can be combined with the regulatory pathway of biofilm formation, thereby improving our understanding of the underlying mechanism of biofilm formation (Brosse et al., 2016).

**Figure 1:**
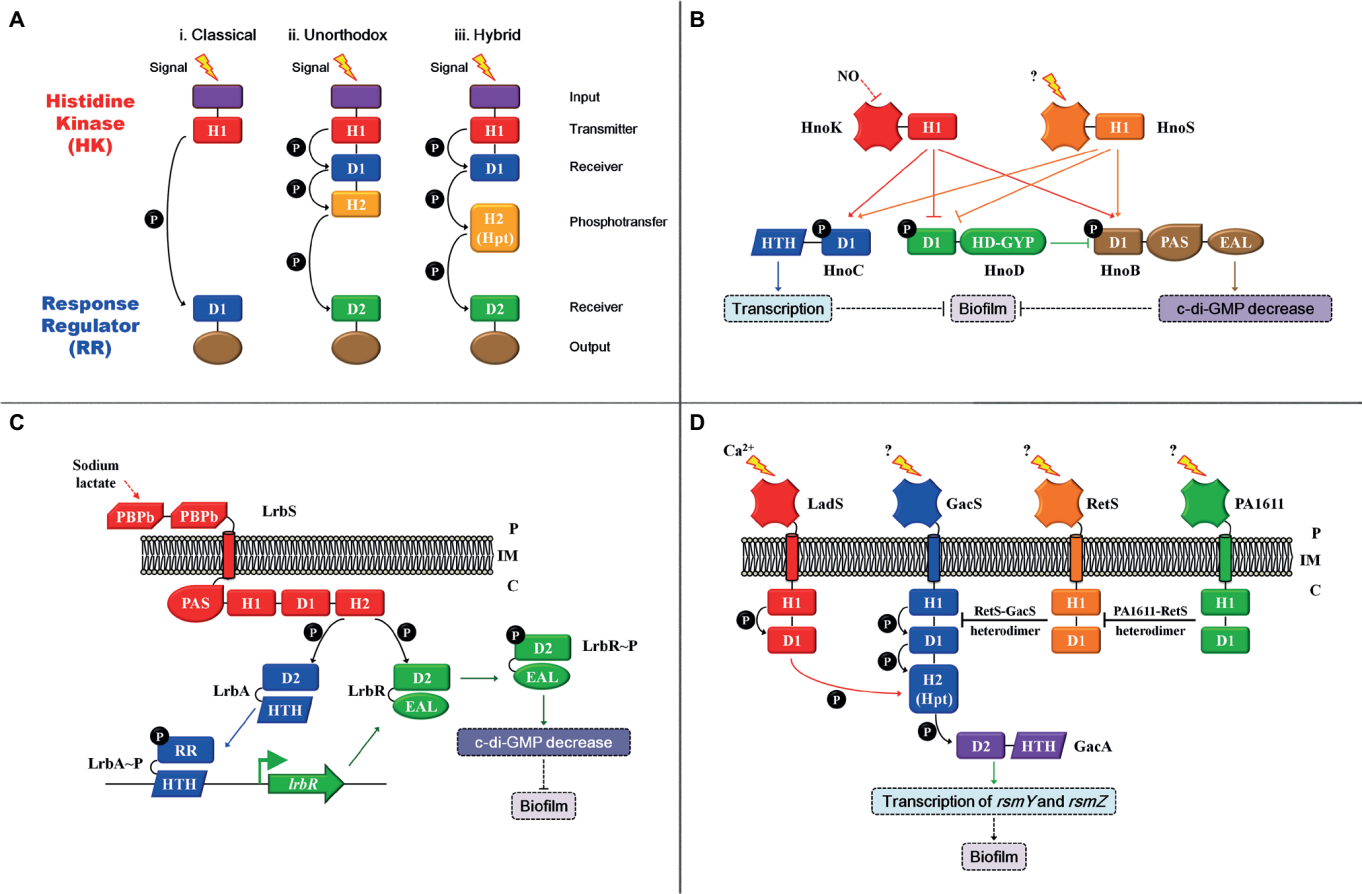
Two-/three-/multi-component signal transduction system modulation patterns. **(A) (i)** The **classical** version comprises an N-terminal input domain (purple), followed by a transmitter (H1) domain (red) with a conserved histidine that can be autophosphorylated in histidine kinase (HK). The phosphoryl group (P) can be transferred to a conserved aspartic residue in the receiver (D1) domain (blue) in the response regulator (RR). The classical version is a two-step phosphorelay mechanism; **(ii)** in the **unorthodox** version, the H1 domain is followed by an additional conserved aspartic residue (D1) and an H2 (yellow) domain in the C-terminal of HK. The phosphoryl group (P) can be transferred to a conserved aspartic residue in the receiver (D2) domain (green) in the RR. The unorthodox version is a four-step phosphorelay mechanism. **(iii)** the **hybrid** version is similar to the **unorthodox** version. The only difference is that the H2 (Hpt) domain of the **hybrid** version is an external phosphotransfer module that acts as an individual protein. **(B)** The modulation mode of the Hno-multi-component signal transduction system. **(C)** The modulation mode of the Lrb-three-component signal transduction system. **(D)** The modulation mode of the Gac-multi-component signaling transduction system; the red arrow indicates that the phosphoryl group (P) can be transferred from the D1 domain of LadS to the H2 domain of GacS. Arrows indicate activation, and the flat end represents inhibition. Solid arrows indicate direct regulation, and dashed arrows represent indirect regulation. Inner membrane (IM), periplasm (P), cytoplasm (C).

TCS is the predominant mode for bacteria to sense and respond to environmental changes (reviewed by Capra and Laub, 2012). It consists of a receptor histidine kinase (HK) and a cognate response regulator (RR). The HK can be divided into three groups according to the mode of its phosphoryl group transfer domains (Figure 1A). In the simplest form, the HK senses a specific signal and then autophosphorylates a conserved histidine residue in the H1 domain. Subsequently, the phosphoryl group is transferred to a conserved aspartic residue in the receiver domain (D1) that is located at the N-terminal of the cognate RR, which is a two-step phosphorelay mechanism and is referred to as the classical version (Figure 1Ai). Unorthodox and hybrid versions of the signal transduction system have also been reported (Figures 1Aii,Aiii). In the unorthodox version, the H1 domain is followed by an additional conserved aspartic residue (D1) and an H2 domain in the C-terminal of HK. Besides, a conserved aspartic residue is referred to the receiver (D2) domain in the RR. The phosphoryl group (P) can be transferred by H1-D1-H2-D2, which is a four-step phosphorelay mechanism. The hybrid version is similar to the unorthodox version, and the only difference is that the H2 (Hpt) domain of the hybrid version is an external phosphotransfer module that acts as an individual protein (reviewed by Capra and Laub, 2012). The two-step phosphorelay mechanism in classical version is a direct and rapid regulatory process. The four-step phosphorelay mechanism in the unorthodox and hybrid versions permits alternative strategies to further fine tune TCS activity, and D1 and H2 domains act as “connecters” that confer regulatory flexibility. For example, HptB is a universal histidine phosphotransfer protein (Hpt) for four HKs, namely, RetS, PA1611, SagS, and ErcS’, which transfer the phosphoryl group from the four HKs to an output RR, HsbR. Such regulatory mode is more economical because it integrates signals sensed by four different HKs to the same output (Lin et al., 2006; Hsu et al., 2008; Bhuwan et al., 2012). Finally, the phosphorylation of RR leads to a conformational change, which activates effector domains and influences the signaling output, such as cellular physiological processes through protein-protein interactions or differential gene expression through protein-deoxyribonucleic acid interactions, thereby mediating the bacteria to adapt to changes in the environment (reviewed by Zschiedrich et al., 2016).

In this mini-review, we provide an overview of some TCSs that mediate biofilm formation in response to specific signals and illustrate their underlying regulatory mechanisms. This information will benefit future investigations on identifying signals and elucidating the underlying mechanisms that induce or inhibit biofilm formation.

## The Two-/Three-/Multi-Component Signal Transduction System

Oxygen signals regulate biofilm formation in various bacterial species, and in several of these bacteria, TCS have been found to transduce oxygen signals (Kolodkin-Gal et al., 2013; Wu et al., 2013). In the plant growth-promoting rhizobacterium, *Bacillus amyloliquefaciens* SQR9, low oxygen levels lead to a reduction in NAD^+^/NADH levels, which are sensed by HK ResE. The activation of HK ResE triggers the transcriptional regulatory activity of its cognate RR ResD, which directly transcribes the *qoxABCD* and *ctaCDEF* operons, thereby synthesizing terminal oxidases (Zhou et al., 2018). These terminal oxidases interact with KinB to activate the core pathway of biofilm formation (Kolodkin-Gal et al., 2013). In the TCS, almost 70% of all classified RRs consist of a DNA-binding domain and function as transcriptional regulators, as previously described (Zschiedrich et al., 2016). Apart from that, some other RRs contain enzymatic output domains that are commonly involved in second messenger homeostasis, such as c-di-GMP, thereby regulating biofilm formation (Zschiedrich et al., 2016). c-di-GMP, a secondary messenger, serves as a core molecule that switches the transition between planktonic growth and biofilm formation in gram-negative bacteria. The current accepted model associates low intracellular levels of c-di-GMP with a planktonic lifestyle, whereas high c-di-GMP levels are associated with biofilm formation. Diguanylate cyclases (DGCs) with conserved GGDEF domains are responsible for c-di-GMP production, whereas phosphodiesterases (PDEs) with conserved EAL or HD-GYP domains are involved in c-di-GMP degradation (reviewed by Dahlstrom and O’Toole, 2017; Jenal et al., 2017). Once the c-di-GMP synthesis and degradation domains are involved in the RR of TCS, the transition between planktonic growth and biofilm formation will directly and exquisitely respond to specific signals.

Nitric oxide (NO) is a highly toxic and reactive compound that can induce biofilm formation in various bacteria, such as *Legionella pneumophila*, *Shewanella oneidensis* MR-1, and *Vibrio cholera* (Carlson et al., 2010; Liu et al., 2012; Plate and Marletta, 2012). In *S. oneidensis* MR-1, NO regulates biofilm formation using a multi-component signal transduction system that involves integration from two HKs, HnoK and HnoS, as well as branching to three RRs: transcriptional factor HnoC, HnoD with degenerate HD-GYP domain, and HnoB with classical EAL domain (Figure 1B). HnoC moderately negatively mediates biofilm formation; HnoB negatively regulates biofilm formation by degrading intracellular c-di-GMP; and HnoD lacks PDE activity but merely suppresses the PDE activity of HnoB *via* direct interactions. Moreover, both HKs, namely, HnoK and HnoS, inhibit biofilm formation by activating HnoC and HnoB. Meanwhile, the two sensory inputs abolish the suppression exerted on HnoB by HnoD. In addition, NO only acts as the signal for HnoK, but no interactions occur between NO and HnoS, indicating that HnoS possibly senses another stimulus (Plate and Marletta, 2012). Although HKs recognize different input signals, both mediate biofilm formation using the same signaling transduction pathway. Investigations on the identification of the input signal of HnoS and why as well as how two different signals are integrated and coordinated to regulate biofilm formation are warranted. In one such example, the PDE RR is not only regulated by HK but also by the other cognate RR. Other examples include a three-component signal transduction system in *Shewanella putrefaciens* CN32. HK LrbS responds to the carbon source sodium lactate and triggers the transcriptional regulatory activity of LrbA by phosphorylation, which subsequently upregulates *lrbR*. Meanwhile, LrbS activates the phosphodiesterase of LrbR, which decreases intracellular c-di-GMP levels, thereby inhibiting biofilm formation (Figure 1C; Liu et al., 2017). The Lrb-three-component and Hno-multi-component signal transduction systems have much more complex regulatory modes in mediating biofilm formation. One of the RRs in both signaling transduction systems is a PDE that associates extracellular-specific signals with the intracellular pool of c-di-GMP, thereby regulating bacterial biofilm formation. Both PDE RRs are modulated by two means. First, activities of both PDE RRs are influenced by the kinase activities of their respective cognate HKs. Moreover, the transcription of the PDE RR gene (*lrbR*) or the PDE activity of the RR HonB is regulated by the other RR (LrbA or HonD) in their signaling transduction systems. In both modulation patterns, the dual control for effector proteins (RR) provides the opportunity to fine-tune the PDE activities. In summary, intracellular c-di-GMP levels are exquisitely and directly regulated by input stimuli of a two-/three-/multi-component signal transduction system, thereby regulating bacterial transition between planktonic existence and biofilm formation.

Biofilm formation in *Pseudomonas aeruginosa* PAO1 is regulated by a multi-component signal transduction system that contains four HKs, namely, RetS, PA1611, LadS, GacS, as well as one RR GacA, by modulating the levels of central small non-coding regulatory RNAs (sRNAs), RsmY and RsmZ (Figure 1D; Chambonnier et al., 2016). In most cases, GacS-GacA is considered as a TCS, and the other three HKs, RetS, PA1611, and LadS, achieve their regulatory function by mediating the core HK GacS rather than controlling the RR GacA directly (Chambonnier et al., 2016). Heterodimer formation between HK RetS and HK GacS impedes GacS kinase activity, thereby preventing phosphorylation of RR GacA (Goodman et al., 2009; Bordi et al., 2010). However, the inhibition on GacS exerted by RetS is released when PA1611 directly binds to RetS (Kong et al., 2013). Furthermore, GasS is an unorthodox HK with an H1-D1-H2 domain (Figures 1Aiii,D), whereas LadS is a hybrid HK with an H1-D1 domain (Figures 1Aii,D). The transfer of a phosphoryl group between both HKs involves an H1_LadS_-D1_LadS_-H2_GacS_-D2_GacA_ signaling pathway. For HK LadS, the H2 domain of GacS is similar to that of an Hpt (H2) individual protein module (Figure 1Aii), and LadS transfers the phosphoryl group to GacA using the H2 of GacS (Chambonnier et al., 2016). In summary, this multi-component signal transduction system has a unique regulatory model: first, although it has four HKs, GacS is the only one that can directly transfer phosphoryl groups to the RR GacA. Moreover, the major modulation pattern depends on the mutual regulation exerted between HKs rather than on RR. In such a regulation model, several different signals can be integrated or coordinated to the central pathway, which is an energy efficient mechanism that is employed by bacteria to adapt and survive amidst environmental changes. Thus far, except for LadS that responds to calcium (Broder et al., 2016), the exact input signals sensed by the other three HKs have not been detected (reviewed by Francis et al., 2017). The identification of the input signals of these HKs is warranted to understand the underlying regulatory mechanism of biofilm formation induced or inhibited by environmental factors. Furthermore, the two HKs, RetS and PA1611, share the universal Hpt with two other HKs, SagS and ErcS’ (Lin et al., 2006; Hsu et al., 2008; Bhuwan et al., 2012). Such regulatory mode integrates various signals, as well as coordinates the multi-component signal transduction system with other regulation pathways, and informs regulation network in bacteria.

## Cross-Regulation

**Figure 2:**
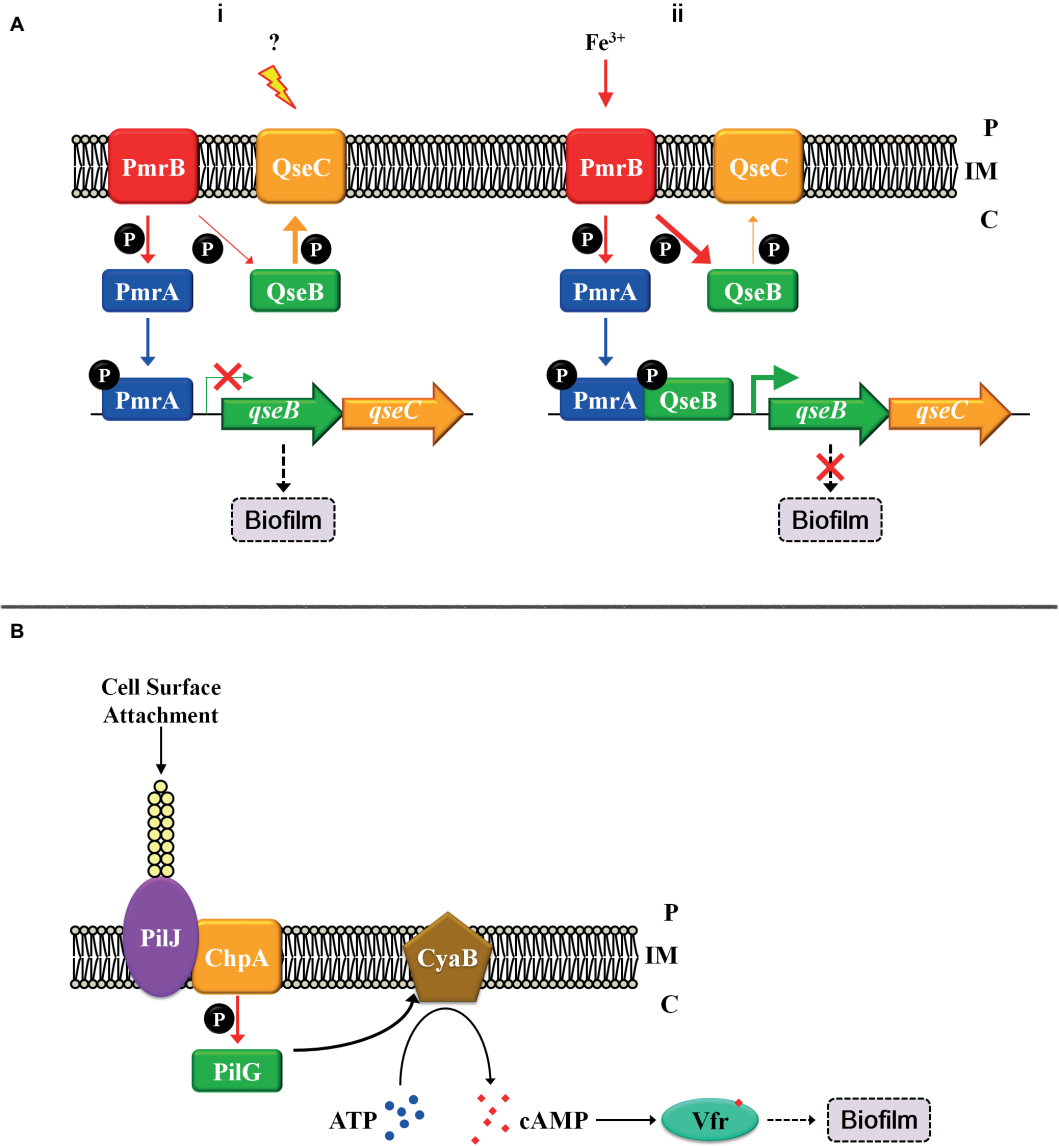
Cross-regulation patterns and the “control system” with TCS. **(A)** Cross-regulation between PmrB-PmrA and QseC-QseB. **(i)** The regulatory pattern in QseC-activated conditions; **(ii)** The regulatory pattern in PmrB-activated conditions. **(B)** Solid surface signal activates TCS ChpA-PilG by TFP. Thick arrows indicate robust regulation, and thin arrows represent weak regulation. Inner membrane (IM). Periplasm (P), cytoplasm (C).

Apart from multi-component signal transduction, cross-talk is another major modulation pattern for the regulation of biofilm formation that integrates and coordinates multiple stimuli. Cross-talk often occurs between two TCSs, and a phosphoryl group is transferred from a HK to a non-cognate RR (reviewed by Goulian, 2010). Rampant cross-talk in cells is harmful to bacteria because this leads to severe confusions, such as the isolation between gene expressions or metabolic changes with their corresponding environmental signals (reviewed by Capra and Laub, 2012). However, appropriate amount of cross-talk may be beneficial for an organism because this may be utilized in diversifying the response to a single input or be integrated with multiple other signals, which is referred to as cross-regulation to distinct from unwanted, disadvantageous cross-talk (reviewed by Laub and Goulian, 2007). Although multiple HKs and RRs are involved in both multi-component signal transduction and cross-regulation pathways, there are huge discrepancies among them. In multi-component signal transduction systems, the transfer of phosphoryl groups from HKs to all RRs occurs at similar rates and is essentially rapidly completed, whereas in cross-regulation, phosphoryl group transfers between cognate pairs occur much more rapidly than that between non-cognate pairs (Laub et al., 2007). Although, in most cases, cross-phosphorylation is relatively weak and slow compared to the regulation between cognate pairs (Yamamoto et al., 2005), some robust cross-regulation has been reported and have biological significance. In uropathogenic *Escherichia coli*, the TCS QseC-QseB has been detected, may respond to quorum sensing, and is involved in pathogenesis and biofilm formation (Bearson et al., 2010; Hadjifrangiskou et al., 2011). The TCS PmrB-PmrA senses ferric irons in the environment and induces *qseBC* transcription (Wösten et al., 2000; Tamayo et al., 2005). A robust cross-regulation occurs between QseC-QseB and PmrB-PmrA. The working model is described as Figure 2A. In the QseC-activated conditions, HK QseC mainly functions to dephosphorylate its cognate partner QseB and prevent QseB from binding to the promoter of its own operon. In such condition, although PmrA∼P can bind to the promoter of *qseBC*, it is not sufficient to transcribe the operon in the absence of QseB, thereby allowing bacteria to engage in a biofilm lifestyle (Figure 2Ai). When the concentration of ferric iron increases in the environment, HK PmrB is excessively activated and phosphorylates QseB more strongly than QseC dephosphorylation. The binding of both RRs PmrA∼P and QseB∼P leads to elevated *qseBC* transcription levels, and the bacteria are allowed to continue their planktonic growth (Figure 2Aii; Guckes et al., 2013). In this example, the states of RR QseB depend on the activity of both HKs or the input stimuli of both HKs. If the input signal of HK QseC increases, then QseB is dephosphorylated by its cognate partner. In contrast, an increase in PmrB input signals leads to the phosphorylation of QseB by this non-cognate HK, and cross-interactions occur. Such example indicates the direct and decisive effect exerted by extracellular stimuli on cellular physiological processes.

## The “Control System” and TCS

Except for the regulation mentioned above, TCSs are also regulated by the “control systems” that integrate TCS with other signal transduction pathways to form a regulatory network *in vivo*. For example, the TCS can be regulated in transcriptional regulation level. In *P. aeruginosa*, TCS FimS-AlgR affects biofilm formation by modulating c-di-GMP synthesis and pili gene expression (Kong et al., 2015), and the transcription of the *fims*-*algR* is directly regulated by the master virulence regulator Vfr (Kanack et al., 2006). This indicates that the TCS FimS-AlgR not only connects signal with biofilm formation but is also regulated by the “control system” and is part of the regulatory network. Solid surface signals are special and important signals that induce starting biofilm formation, and the TCS ChpA-PilG is involved into responding to such signals. As it is difficult for HK ChpA to directly sense the solid surface signals, the “control system” type IV pilus (TFP) is involved to connect both. When *P. aeruginosa* comes into contact with a solid surface, attachment and retraction exerts a change in tension of TFP, and the TFP chemosensory protein PilJ transduces the attachment signal to the cytoplasm by directly interacting with the HK ChpA (Persat et al., 2015). The TCS ChpA-PilG subsequently stimulates the adenylate cyclase CyaB, leading to an increase in cellular cAMP concentration, thus activating Vfr that promotes biofilm formation *via* multiple pathways (Figure 2B; Fulcher et al., 2010). This finding indicates that TFP acts as a mechanosensory “control system” that activates TCS ChpA-PilG. Besides, TFP can activate other signal transduction pathways, such as FimS-AlgR (Luo et al., 2015), which connects these pathways and integrates them into a regulatory network. This suggests again that a “control system” coordinates and integrates TCSs or other transduction pathways to be a regulatory network.

In *Bacillus subtilis*, HK DegS is required in the transition from planktonic cell to surface-attached biofilm (reviewed by Belas, 2014). However, how cytoplasmic HK DegS senses the attachment is unknown. It is conceivable that some cytoplasmic flagellar components may directly interact with DegS and stimulate its activity, similar to the interaction between PilJ and ChpA. The above examples show that multiple “control systems” modulate the expression and activity of TCSs, thus establishing a complicated network that precisely regulates physiological processes.

## Conclusions and Perspectives

Biofilm formation is an important bacterial lifestyle that allows rapid adaptation to adverse environments. TCS is a key strategy for bacteria to monitor environmental or internal signals and translate these stimuli into appropriate cellular responses. Thus far, TCS is the main pathway involved in bacterial biofilm formation, and extensive investigations have been conducted to date. However, there are still certain issues that need to be resolved. First, the environment is relatively complex, thus, different input signals sensed by these TCSs are difficult to find. Identifying more input signals of TCSs may provide critical cues for biofilm formation. Moreover, in most identified TCSs, although the simple communications between the cognate pairs or the cellular physiological processes controlled by these TCSs have been investigated, more complex underlying mechanisms may exist. For example, cross-regulation may occur among some TCSs, which may be combined to mediate the same physiological process, or complicated modulation models may exist between TCSs with other signaling transduction systems, which may integrate and coordinate multiple signaling transduction networks. Biofilm formation is complex and exquisitely regulated by various physiological processes. Investigations on the input signals and mechanisms of TCSs benefit to identify more signals clues and signaling transduction pathways involved in biofilm formation. Finally, when multiple signals are integrated to mediate the downstream physiological processes by the same multi-component signal transduction or a cross-regulation pathway, the relationship between these signals, why these signals are integrated in evolution, and whether restriction occurs among them should be examined. Identifying the restricted or promoted relationship between these signals is also necessary to further understand biofilm formation and its regulation. In summary, the transition from planktonic growth to biofilm is important for bacteria survival in natural environment, and plentiful genes expression and cellular physiology processes are changed during the transition. TCSs are critical for regulating these processes. Cross-regulation and the regulation by “control system” promote the coordinate regulation between TCSs with some other transduction pathways. Besides, the multi-component signal transduction systems contain more than one HKs and RRs that can respond to more signals and interact with other regulation pathways. TCSs are the “connecter” and “core” of the regulatory network in bacteria, which promote the lifestyle transition well-organized by integrating different signals and coordinating multiple regulation pathways.

## Author Contributions

CL, DS, and JZ conducted the literature study and wrote the draft manuscript. WL edited and revised the manuscript.

### Conflict of Interest Statement

The authors declare that the research was conducted in the absence of any commercial or financial relationships that could be construed as a potential conflict of interest.
